# Tumor cell expression of MMP3 as a prognostic factor for poor survival in pancreatic, pulmonary, and mammary carcinoma

**DOI:** 10.18632/genesandcancer.90

**Published:** 2015-11

**Authors:** Christine Mehner, Erin Miller, Aziza Nassar, William R. Bamlet, Evette S. Radisky, Derek C. Radisky

**Affiliations:** ^1^ Department of Cancer Biology, Mayo Clinic, Jacksonville, FL, USA; ^2^ Department of Pathology, Mayo Clinic, Jacksonville, FL, USA; ^3^ Department of Health Sciences Research, Mayo Clinic, Rochester, MN, USA

**Keywords:** MMP3, IHC, breast cancer, lung cancer, pancreatic cancer

## Abstract

Breast, lung, and pancreatic cancers collectively represent one third of all diagnosed tumors and are responsible for almost 40% of overall cancer mortality. Despite improvements in current treatments, efforts to develop more specific therapeutic options are warranted. Here we identify matrix metalloproteinase 3 (MMP3) as a potential target within all three of these tumor types. MMP3 has previously been shown to induce expression of Rac1b, a tumorigenic splice isoform of Rac1. In this study we find that MMP3 and Rac1b proteins are both strongly expressed by the tumor cells of all three tumor types and that expression of MMP3 protein is prognostic of poor survival in pancreatic cancer patients. We also find that MMP3 gene expression can serve as a prognostic marker for patient survival in breast and lung cancer. These results suggest an oncogenic MMP3-Rac1b signaling axis as a driver of tumor progression in three common poor prognosis tumor types, further suggesting that new therapies to target these pathways could have substantial therapeutic benefit.

## INTRODUCTION

Lung cancer is the leading cause of death for both sexes with a 221,000 new cases and 158,000 deaths estimated for 2015 in the US [1]. Breast cancer has the highest incidence and remains the second highest cause of death for women in the US, with an estimated 232,000 new cases and 40,000 deaths in 2015. Pancreatic cancer, though rarer in incidence, with estimated 49,000 new cases in 2015, ranks fourth overall in cancer related deaths for both sexes, with an estimated 40,500 deaths in 2015. Combined, these three cancer types represent nearly one third of all new tumor diagnoses and cause more than 40% of the cancer related deaths in the United States. While progress has been made to develop new methods of early detection [2, 3] and improved treatment [4–7] for these cancer types, better and more specific therapeutic options are still needed.

Investigations of the processes involved in cancer development and tumor metastasis have identified matrix metalloproteinases (MMPs) as key factors involved in the development of the tumor microenvironment and as drivers of cancer progression and metastasis [8–10]. These findings generated significant enthusiasm for MMPs as therapeutic targets, but clinical trials that employed broad spectrum, small molecule catalytic site inhibitors produced disappointing results [11]. In Phase III studies, the broad spectrum MMP inhibitor marimastat failed to extend progression-free survival of metastatic breast cancer patients [12], the broad spectrum MMP inhibitor prinomastat did not affect overall survival or time to progression for non small-cell lung cancer patients [13], and the broad spectrum MMP inhibitor BAY 12- 9566 failed to improve progression-free survival for metastatic adenocarcinoma of the pancreas [14]. While the pharmaceutical industry has been hesitant to further explore MMP inhibitors as anticancer therapeutics following these trials, ongoing basic research suggests that more selective MMP inhibitors with lower toxicity could be achievable, and would likely produce better results, if targeted toward specific MMPs that are upregulated in human cancers and that drive malignant progression [15].

MMPs are a family of 24 enzymes, some members of which are easier to detect by zymography methods and have been studied more extensively in the context of cancer progression and metastasis, while other members of the MMP family are more difficult to visualize. MMP3, classified as a stromelysin for its ability to cleave a variety of extracellular matrix protein substrates, is an example of an MMP family member that is more challenging to detect, and consequently has not been as widely studied as a potential biomarker for cancer prognosis as many other MMPs. While many studies using cultured cells or animal models have implicated MMP3 as a functional contributor to lung, breast, and pancreatic premalignancy and cancer [16–23], much less is known about how MMP3 expression in human tumors relates to disease progression and overall survival.

In this study, we evaluate the stromal and epithelial cell expression of MMP3 in lung, breast, and pancreatic cancer. We extend our findings from a previously described tissue microarray study consisting of patients with pancreatic adenocarcinoma [18], and integrate analyses of tissue biopsies and annotated datasets derived from lung and breast cancer patients. Our results reveal the importance of tumor cell expression of MMP3 in all three of these tumor types for tumor progression and overall survival. Taken together with prior functional studies of MMP3 in experimental models of these cancers, our findings suggest that MMP3 may offer a viable target for therapy relevant to multiple cancer types that account for a large proportion of cancer mortality.

## RESULTS

### MMP3 is selectively expressed in pancreatic adenocarcinoma tumor cells and is prognostic of patient survival

**Figure 1:**
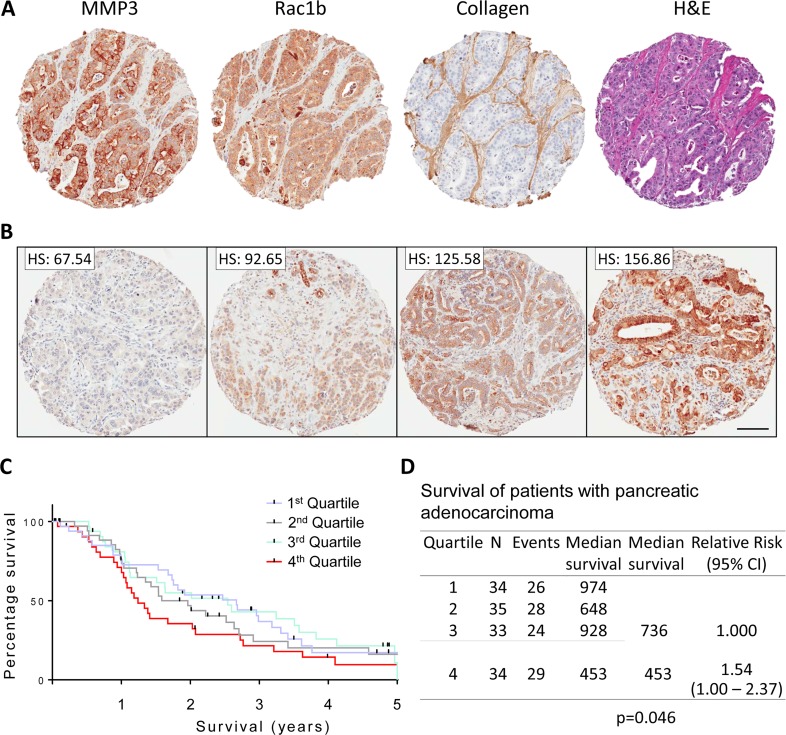
MMP3 and Rac1b tissue expression in pancreatic adenocarcinoma (A) Representative biopsy tissue spots stained with MMP3, Rac1b, collagen-I, and H&E showing clear staining signal within the tumor cells for MMP3 and Rac1b and stromal staining for collagen-I. (B) H-score generation and distribution of staining intensity into quartiles. Scale bar = 100um (C) Kaplan-Meier survival analysis among the patients separated into quartiles. (D) Survival analysis comparing low MMP3 expressing quartiles 1-3 and high MMP3 expressing quartile 4.

Pancreatic adenocarcinoma tissue microarrays (TMAs) were stained for MMP3, Rac1b (a tumorigenic splice isoform of Rac1 previously shown to be upregulated by MMP3 in pancreas, breast, and lung cancers [18, 20, 21]), collagen I, and H&E. Using collagen-1 as a marker of stromal tissue, we observed that MMP3 and Rac1b were primarily expressed in the pancreatic tumor cells (Figure 1A). Using the TMA-lab analysis software, we determined staining intensity and distribution for each tissue spot, generating an H-Score (scoring 0-300). In a previous study, we identified a significant correlation between MMP3 expression and tumor grade, where grade IV tumors showed the highest intensity staining [18]. We now evaluated the association of MMP3 staining intensity with patient prognosis following biopsy. When dividing the patients into quartiles according to tumor MMP3 expression intensity (Figure 1B, representative staining intensities), we observed an apparent distinction between the lower three quartiles and the fourth (Figure 1C). When we compared low (quartiles 1-3) to high (quartile 4) expression, we found median survival of 736 days for the lower three quartiles, while the fourth quartile had a median survival of 453 days (p=0.046, Figure 1D). These results warrant the investigation of MMP3 as a prognostic tool for pancreatic cancer patients, although validation of these findings in larger cohorts will be required.

### MMP3 shows selective expression in breast and lung carcinoma cells

**Figure 2:**
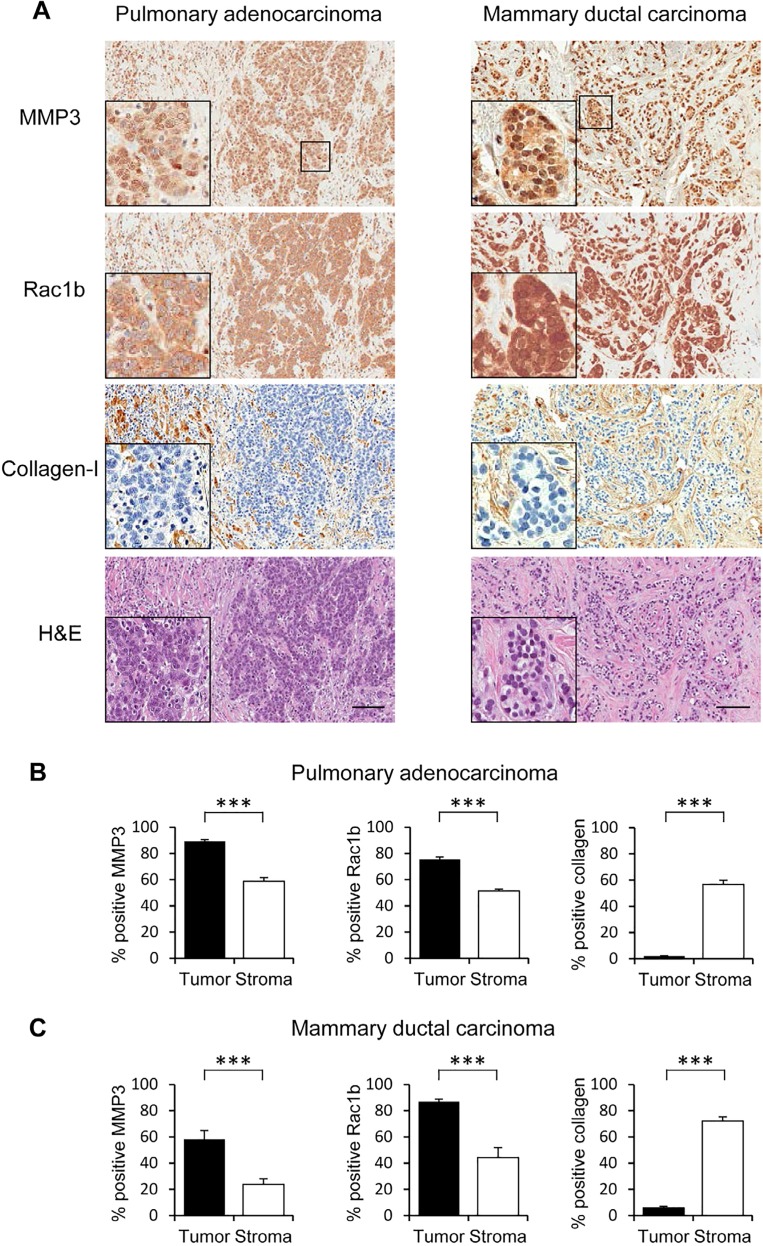
MMP3 and Rac1b tissue expression in pulmonary adenocarcinoma and mammary ductal carcinoma Stains for MMP3 and Rac1b show localization in tumor cells; collagen-I demarcates regions of stroma. Scale bar = 100um. Comparing staining intensity using H-score (scale 0-300). (B, C) Staining intensity for MMP3, Rac1b, and collagen-I, reported is an average of six 100um2 slide views, (B) in lung adenocarcinoma with significantly higher intensity in the tumor tissue for MMP3 and Rac1b and higher intensity in the stroma for collagen-I staining. (C) The mean of MMP3 and Rac1b expression intensities are significantly higher in the tumor tissue in contrast to high collagen-I levels in the stroma. (*** p<0.0001 (unpaired t-test), error bars SEM).

Patient breast and lung cancer tissue biospecimens revealed epithelial staining patterns for MMP3 (Figure 2A), similar to pancreatic carcinoma TMAs (Figure 1A). In both lung and breast cancer biospecimens, we found MMP3 staining primarily in the tumor cells, with much less MMP3 staining in the surrounding stroma (lung: Figure 2B left panel; breast: Figure 2C left panel). Rac1b was also analyzed in these tissue samples and showed a similar expression pattern in which staining was primarily found in the cancer cells (lung: Figure 2B center panel; breast: Figure 2C center panel). Thus, both MMP3 protein and its downstream mediator Rac1b are highly expressed in cancer cells in these three tumor types.

### MMP3 expression is prognostic of outcome in breast cancer

**Figure 3:**
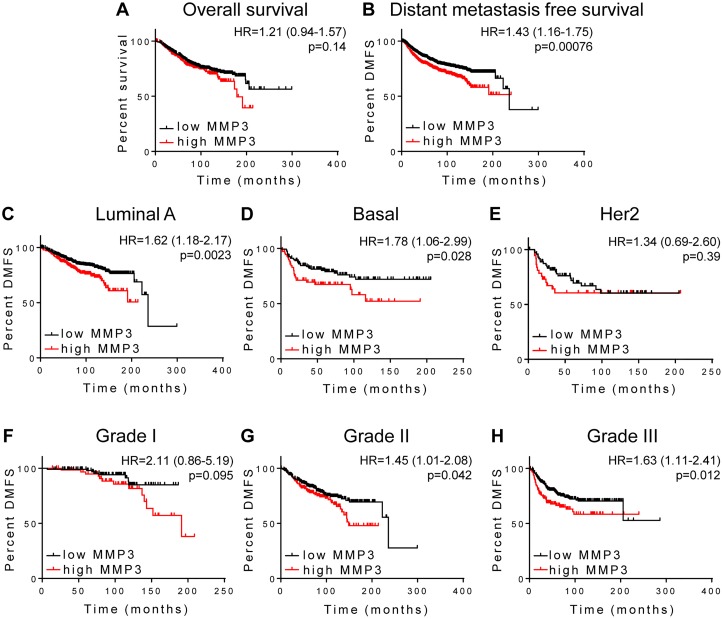
MMP3 association with outcome in breast carcinoma Kaplan-Meier survival analysis using KM-plotter was used to analyze MMP3 association with (A) overall survival (N=1117) and (B-H) distant metastasis free survival (DMFS). (B) Full cohort, N=1609; (C) Luminal A tumors, N=918; (D) Basal tumors, N=219; (E) Her2 positive tumors, N=111; (F) grade I, N=172; (G) grade II, N=495; (H) grade III, N=391.

We used the KM Plotter web utility (25) to analyze gene expression in breast cancer patients using endpoints of overall survival (OS), finding that patients with increased MMP3 did not show significantly different outcome (Figure 3A; N=1117, p=0.14). However, when considering distant metastasis-free survival (DMFS) as endpoint, we found that patients expressing higher levels of MMP3 had a significantly poorer outcome (Figure 3B; N=1609; HR=1.43 [95% CI 1.16-1.75], p=0.00076). When patients were segregated into cohorts according to intrinsic tumor subtypes, we found that high MMP3 expression was significantly associated with poor DMFS for patients with luminal A subtype tumors (Figure 3C, N=918; HR=1.6 [95% CI 1.18-2.17], p=0. 0023) and with basal subtype tumors (Figure 3D, N=219; HR=1.78 [95% CI 1.06-2.99], p=0.028). Association of MMP3 expression with DMFS was not significant in the HER2-positive subtype cohort (Figure 3E, N=111, p=0.39). We also found that the association of MMP3 expression with survival became stronger with increasing tumor grade: while grade I tumors showed a nonsignificant association with MMP3 (Figure 3F, N=172, p=0.095), we found progressive increases in hazard ratio and significance for grade II (Figure 3G, N=495, HR=1.45 [95% CI 1.01-2.08], p=0.042) and grade III tumors (Figure 3H, N=391, HR=1.63 [95% CI 1.11- 2.41], p=0.012).

### MMP3 expression is prognostic of outcome in lung adenocarcinoma

**Figure 4:**
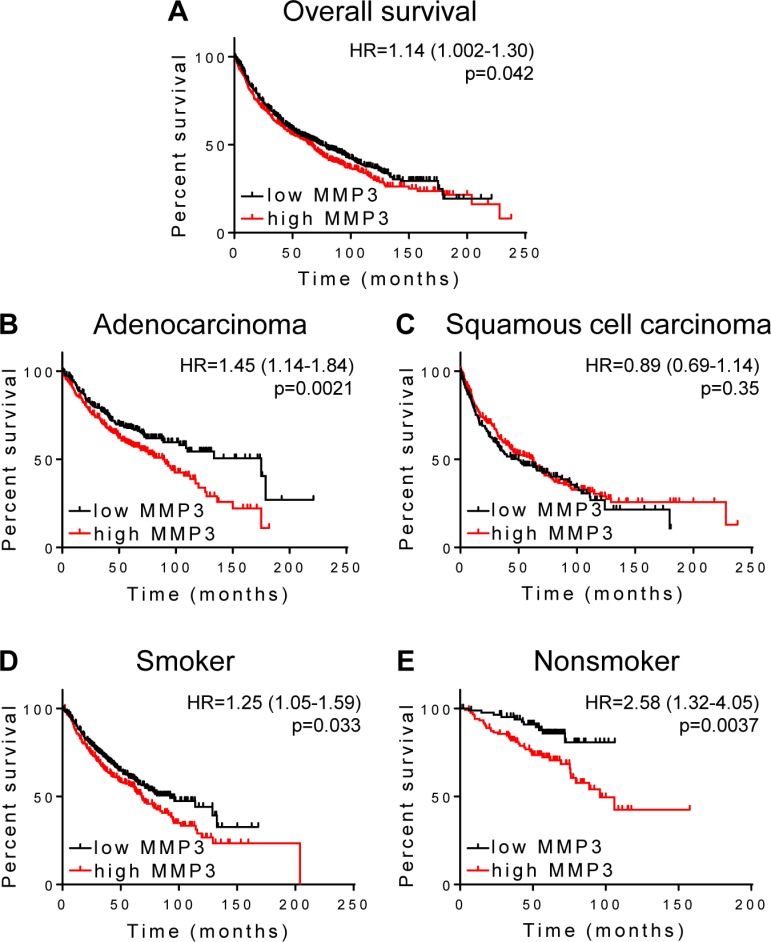
MMP3 association with survival in lung cancer Kaplan-Meier survival analysis using KM-plotter was used to analyze MMP3 association with overall survival in patients with (A) pulmonary carcinoma, N=1926; (B) adenocarcinoma, N=720; (C) squamous cell carcinoma, N=524; (D) smokers, N=820; (E) nonsmokers, N=205.

We next used the KM Plotter web utility to similarly analyze gene expression in lung cancer patients using OS as the endpoint; for this data set DMFS data were not available. Elevated MMP3 expression in lung tumors was significantly associated with poorer OS in the full cohort (Figure 4A, N=1926, HR=1.14 [95% CI 1.002-1.30], p=0.042). Individual analyses of cohort subsets representing the two major histological subtypes, adenocarcinoma and squamous cell carcinoma, revealed that tumor MMP3 expression is more strongly associated with poor survival in patients with lung adenocarcinoma (Figure 4B, N=720, HR=1.45 [95% CI 1.14-1.84], p=0.0021), while no significant association was found in patients with squamous cell carcinoma (Figure 4C, N=524, p=0.35). The subset of smokers shows a significant association of MMP3 tumor expression with OS (Figure 4D, N=820, HR=1.25 [95% CI 1.05-1.59], p=0.033), while an even stronger association of tumor MMP3 expression with poor OS is found in analysis of the patient subset of nonsmokers (Figure 4E, N=205, HR=2.58 [95% CI 1.32-4.05], p=0.0037). Overall we conclude that MMP3 is prognostic for poor survival in lung adenocarcinoma, with particularly strong association with outcome in nonsmokers.

## DISCUSSION

In this study we show that MMP3 and its downstream effector Rac1b are both strongly expressed specifically in tumor cells in pancreatic, breast, and lung carcinomas, and that tumor expression of MMP3 is correlated with poor patient survival and earlier recurrence in all of these cancers. In our previous work, we have elucidated a tumorigenic signaling pathway wherein MMP3 induces expression of Rac1b, a constitutively active splice isoform of the small GTPase Rac1, leading to induction of epithelial mesenchymal transition (EMT) and genomic instability [20, 26]. Using transgenic mouse models and cultured cells, we and others have found that this signaling axis can drive malignant transformation and progression of breast, lung and pancreatic cancers [18, 20, 21]. Importantly, the present study provides support for the clinical relevance of the oncogenic MMP3-Rac1b signaling axis in all three of these tumor types, (a) by demonstrating co-localized staining for MMP3 and Rac1b in tumor tissue, and (b) by revealing association of tumor MMP3 expression with poor outcome of patients with these three tumor types. In aggregate, our studies suggest that this pathway offers opportunities for therapeutic intervention in breast, lung, and pancreatic cancers, three common and aggressive cancers that represent a large proportion of cancer mortality. It is further possible that the mechanisms we have defined in these cancers are also of relevance to additional cancers, as in addition to the implicated role of Rac1b in the development of breast [16, 20, 21, 27–32], lung [21, 32–34], and pancreatic [18, 35, 36] cancer development and progression, Rac1b has also been implicated in colorectal [37–51], ovarian [52], and papillary thyroid [53] cancers.

One unexpected finding of our study is the localization of MMP3 primarily to tumor cells; this is somewhat surprising since MMPs have been generally believed to derive mainly from the tumor stroma [54], where they have been found to play important roles in shaping the tumor microenvironment [8, 9, 23]. Importantly, the cell type of origin has been found previously to be a significant factor modulating prognostic interpretation for a number of MMPs in breast cancer [55], including MMP3 specifically [56, 57]. By revealing MMP3 staining to be strongly associated with epithelial tumor cells, our present findings suggest an autocrine MMP3-Rac1b signaling mechanism is involved in malignant progression of breast, lung, and pancreatic cancers. It should be noted that while the antibody used for the MMP3 IHC study does not distinguish between zymogen or active forms of the enzyme, our previous work has shown that the catalytic activity of MMP3 is necessary for induction of Rac1b [20, 58, 59], so it is likely that a substantial fraction of the MMP3 is present in the active form.

Our identification of an oncogenic MMP3-Rac1b signaling axis as a driver of tumor growth and progression of multiple common poor prognosis cancers suggests that new therapies to target this pathway may hold substantial clinical promise. Direct targeting of Rac1b is anticipated to be challenging, since like other small GTPases [60, 61], its pleiotropic activities are mediated through diverse protein-protein interactions, and as such may be difficult to target pharmacologically. Nevertheless, efforts to identify the protein effectors preferentially recruited by Rac1b and responsible for its distinct phenotypic program [30, 41, 58], and to biologically and structurally characterize these interactions, may lead to opportunities to disrupt these interactions for therapeutic benefit [62]. Key epitopes involved in these protein-protein interactions may potentially be targeted by therapeutic antibodies; alternatively, recent years have seen impressive advances in drug development programs using fragment-based approaches to identify small molecule therapeutic inhibitors of protein-protein interactions [60, 63]. Alternatively, Rac1b function could be targeted using existing therapeutic agents. EHT1864 is an inhibitor of Rac1 family GTPases which has been reported to have selectivity toward Rac1b [64, 65] and which has been found to inhibit estrogen-dependent breast cancer cell proliferation in culture models [66]. Other possibilities include the natural products sanguinarine [67] and the anti-inflammatory drugs ketorolac [52] and ibuprofen [50], which also have been shown to inhibit Rac1b function, although the effects of these compounds as anticancer therapeutics in clinical settings remains to be evaluated.

As the extracellular initiator of the oncogenic pathway, MMP3 may offer an even more tractable molecular target for drug development. While early oncology trials of nonselective small molecule MMP inhibitors proved disappointing [11–13], it is likely that better results could be achieved by selective targeting of specific MMPs that contribute to tumor growth and malignant progression; our studies in breast, lung, and pancreatic cancer suggest that MMP3 may be a good candidate. Achieving selectivity with small molecule MMP inhibitors has proven very challenging, but a number of recent advances may help to make development of selective MMP-3 inhibitors achievable [15]. In particular, while similarities in MMP active sites make discrimination by small molecule inhibitors problematic (68), greater selectivity can potentially be achieved by exploiting insights from structural analyses and targeting less conserved epitopes using antibodies or engineered forms of the natural tissue inhibitors of metalloproteinases (TIMPs) [15, 69–72].

If drugs can be developed to intervene in the oncogenic MMP3-Rac1b signaling axis, a parallel challenge will be to identify the patients most likely to respond to this therapeutic strategy. The present study suggests that some cancer subtypes may be more responsive than others, since, for example, MMP3 expression was significantly prognostic of outcome in lung adenocarcinoma but not in squamous cell carcinoma; further studies to validate our observations in additional clinical cohorts of breast, lung, and pancreatic cancers are warranted. Ultimately, implementation of a therapeutic strategy to intervene in the MMP3-Rac1b signaling axis may be best guided using companion biomarkers. Our studies to date of a large cohort of pancreatic cancer patients suggest that MMP3 staining may be useful as a prognostic tissue biomarker, and might be anticipated to predict response to therapies targeting this axis.

## MATERIALS AND METHODS

### Pancreas TMA and immunohistochemistry

Pancreatic adenocarcinoma formalin-fixed, paraffin-embedded (FFPE) tissue samples mounted as a tissue microarray (TMA) were obtained through the Mayo Clinic Pancreas Cancer SPORE. Each TMA slide contains up to 432 cores representing each patient with three spots (n=140) and 12 process controls. TMA slides were stained for human MMP3 (ProteinTech #17873- 1-AP, dilution 1:100), human Rac1b (Millipore #09- 271, dilution 1:1500), and human Collagen-I (Abcam #ab34710, dilution 1:5000). TMAs were deparaffinized and rehydrated in graded alcohol into water. Antigen retrieval was done in citrate buffer pH 6.0 for 25min at 100C. Followed by 3% H2O2 treatment for 5min and serum free protein block for 5min. Slides were then stained for 1 h at room temperature with the respective antibodies. Followed by 30 min with secondary anti-rabbit labeled polymer/horse radish peroxidase conjugate (Dako #K4003) and then color was developed for 5min using 3,3′-diaminobenzidine (DAB, EnVision+, Dako). Slides were counterstained with hemotoxin. The stained slides were analyzed using Image Scope Software application TMA-lab (Image Scope Software, Aperio Technologies) with a color deconvolution. The stained slides were analyzed using Image Scope Software application TMA-lab (Image Scope Software, Aperio Technologies) with a color deconvolution algorithm that generates an H-score (scale 0-300) to allow non-biased interpretation of intensity and positivity of patient samples (score is acquired as follows: 1.0x(%weak positive)+2.0x(%median positive)+3.0x(%strong positive)). This scoring method has been widely used to objectively quantify receptor expression and different stains in lung, ovarian and prostate cancers [24]. Spot scoring and staining distribution analysis as performed by the investigators were blinded to patient characteristics and patient outcome. Samples were grouped in quartiles based on the H-score and analyzed as quartile 1 (Q1), quartiles 2 (Q2; quartile 3 (Q3), and quartile 4 (Q4).

Additional breast cancer and lung cancer human tissue samples were provided by Mayo Clinic Jacksonville Department of Cancer Biology histology and stained for human MMP3, human Rac1b, and human Collagen-I. The slides were subsequently analyzed for tumor and stroma staining and staining patterns for the individual markers were determined.

### Statistical analysis

Average patient expression level was calculated by taking the mean of the multiple core level H-scores for an individual. Overall survival was compared across quartiles of mean patient expression level using Kaplan-Meier methods and log-rank tests. A post-hoc analysis combining quartiles 1-3 compared to quartile 4 was considered to explore differences in survival for the subset of patients with the highest mean expression. Cox Proportional Hazards regression models were utilized to estimate Hazard Ratios and 95% Confidence Intervals. Statistical analysis related to the TMA scoring and relevant patient characteristics was performed using SAS/STAT software, version 9.2 of the SAS System for UNIX.

The Kaplan-Meier Plotter results were obtained using the current release of Kaplan Meier Plotter (http://www.kmplot.com; [25]) using the 09/2015 update containing 4,142 patients for breast and 2,437 patients for lung cancer, using Affymetrix ID: “205828_at”, survival set at distant metastasis-free survival or overall survival, as indicated, auto select best cutoff set as checked, follow-up threshold set at all, and array quality control set at exclude biased arrays.
